# An automated method to discover true events and classification of intracellular Ca^2+^ profiles for endothelium *in situ* injury assay

**DOI:** 10.3389/fphys.2023.1161023

**Published:** 2023-05-11

**Authors:** Marcial Sánchez-Tecuatl, Francesco Moccia, Jorge F. Martínez-Carballido, Roberto Berra-Romani

**Affiliations:** ^1^ Electronics Department, Instituto Nacional de Astrofísica, Óptica y Electrónica, Puebla, Mexico; ^2^ Laboratory of General Physiology, Department of Biology and Biotechnology “Lazzaro Spallanzani”, University of Pavia, Pavia, Italy; ^3^ Department of Biomedicine, School of Medicine, Benemérita Universidad Autónoma de Puebla, Puebla, Mexico

**Keywords:** automated method, intracellular Ca^2+^, endothelium, true event identification, signal feature, signal processing

## Abstract

**Introduction:** Endothelial cells (ECs), being located at the interface between flowing blood and vessel wall, maintain cardiovascular homeostasis by virtue of their ability to integrate chemical and physical cues through a spatio-temporally coordinated increase in their intracellular Ca^2+^ concentration ([Ca^2+^]i). Endothelial heterogeneity suggests the existence of spatially distributed functional clusters of ECs that display different patterns of intracellular Ca^2+^ response to extracellular inputs. Characterizing the overall Ca^2+^ activity of the endothelial monolayer in situ requires the meticulous analysis of hundreds of ECs. This complex analysis consists in detecting and quantifying the true Ca^2+^ events associated to extracellular stimulation and classifying their intracellular Ca^2+^ profiles (ICPs). The injury assay technique allows exploring the Ca^2+^-dependent molecular mechanisms involved in angiogenesis and endothelial regeneration. However, there are true Ca^2+^ events of nearly undetectable magnitude that are almost comparable with inherent instrumental noise. Moreover, undesirable artifacts added to the signal by mechanical injury stimulation complicate the analysis of intracellular Ca^2+^ activity. In general, the study of ICPs lacks uniform criteria and reliable approaches for assessing these highly heterogeneous spatial and temporal events.

**Methods:** Herein, we present an approach to classify ICPs that consists in three stages: 1) identification of Ca^2+^ candidate events through thresholding of a feature termed left-prominence; 2) identification of non-true events, known as artifacts; and 3) ICP classification based upon event temporal location.

**Results:** The performance assessment of true-events identification showed competitive sensitivity = [0.9995, 0.9831], specificity = [0.9946, 0.7818] and accuracy = [0.9978, 0.9579] improvements of 2x and 14x, respectively, compared with other methods. The ICP classifier enhanced by artifact detection showed 0.9252 average accuracy with the ground-truth sets provided for validation.

**Discussion:** Results indicate that our approach ensures sturdiness to experimental protocol maneuvers, besides it is effective, simple, and configurable for different studies that use unidimensional time dependent signals as data. Furthermore, our approach would also be effective to analyze the ICPs generated by other cell types, other dyes, chemical stimulation or even signals recorded at higher frequency.

## 1 Introduction

Changes in the intracellular Ca^2+^ concentration ([Ca^2+^]_i_) control a wide array of functions across the phylogenetic tree, ranging from proliferation to apoptosis (Carafoli, 2002). The endothelium is a monolayer of interconnected cells that form the innermost layer of blood vessels and is in direct contact with blood flow, thereby playing a crucial role in the proper functioning of the cardiovascular system. The spatiotemporal diversity of [Ca^2+^]_i_ signals underpins virtually all endothelial cell functions (McCarron et al., 2017), including angiogenesis (Negri et al., 2019), barrier permeability (Genova et al., 2020), vasomotion (Negri et al., 2021), and gene expression (Zhu et al., 2008). Accordingly, the deregulation of endothelial Ca^2+^ signaling may lead to life-threatening disorders, such as diabetes mellitus, peripheral artery disease, heart failure, and cancer (Ding and Triggle, 2010; Berra-Romani et al., 2012; Dixit and Simon, 2012; Favero et al., 2014; Guerrero-Hernandez and Verkhratsky, 2014; Chaudhari and Ma, 2016; Keller et al., 2017; Moccia et al., 2020).

Being located at the interface between blood stream and intraparenchymal tissues, endothelial cells are continuously subjected to pulsatile stretch, laminar shear stress, and changes in extracellular matrix stiffness (Zeng et al., 2022). Mechanical cues stimulate ECs to release a plethora of vasoactive and inflammatory mediators, growth factors, and reactive oxygen species (Cahill and Redmond, 2016). Pathological conditions, such as atherosclerosis and hypertension, impair the endothelial response to hemodynamic forces, thereby leading to endothelial dysfunction, atherosclerotic plaque formation, and ultimately, plaque rupture. Endothelial disruption is also caused by clinical catheter-based interventional strategies, where the therapeutic outcome could be hampered by the subsequent inflammatory response and loss of endothelial integrity. Understanding the regulatory mechanisms leading to endothelial regeneration is, therefore, required to interfere with endothelial dysfunction and maintain cardiovascular homeostasis (Cornelissen and Vogt, 2019). To do so, it is mandatory to dissect the complex Ca^2+^ activity that is triggered by mechanical injury both at the lesion edge and at more remote sites (Berra-Romani et al., 2008; Zhao et al., 2008; Berra-Romani et al., 2012; Berra-Romani et al., 2013).

Many efforts have been devoted to elucidate the bewildering complexity of the endothelial Ca^2+^ waves and underlying signal transduction mechanisms (McCarron et al., 2017). Most of these studies were based on the two-dimensional representation of a sequence of consecutive measurements of the changes in the fluorescence of Ca^2+^-sensitive dyes that reflect variations in [Ca^2+^]_i_, also known as their intracellular Ca^2+^ profile (ICP). Currently, most measurements are intensity averages of defined regions of interests (ROIs) drawn around cells loaded with a Ca^2+^-sensitive fluorophore, at a sampling rate that depends on the frequency or rate of increase of the Ca^2+^ signal.

The availability of better computational resources and the development of algorithms that help the extraction (Shah et al., 2020) and analysis of ICPs (Francis et al., 2012) have provided a new perspective in the study of endothelial Ca^2+^ signaling at the tissue level. Collaborative sensory networks have recently been reported in the vascular endothelium exposed to multiple chemical cues (Cahill and Redmond, 2016; Cornelissen and Vogt, 2019), and a remarkable heterogeneity in the Ca^2+^ response to different agonists has emerged (David and Lambert, 2013; Wilson et al., 2016; McCarron et al., 2017). Tissue-level studies require temporal analysis of multiple (∼hundreds) ICPs and careful evaluation of the structural characteristics of responding cells to detect the variations that represent a true Ca^2+^ event. The proper detection of true Ca^2+^ events allows a subsequent classification and characterization of the Ca^2+^ responses at the tissue level.

Some challenges must be overcome since most of the Ca^2+^ signals evoked by chemical and mechanical stimuli present a very small magnitude and are superimposed on a baseline [Ca^2+^]_i_ that is noisy so that physiological Ca^2+^ responses are often too difficult to be solved as discrete Ca^2+^ events during fluorescence recordings (Lee et al., 2018). Mechanical stimulation, such as that imposed by endothelial scraping to investigate Ca^2+^ activity at the wound edge (Berra-Romani et al., 2008; Zhao et al., 2008; Berra-Romani et al., 2012; Berra-Romani et al., 2013), introduces additional undesirable artifacts, such as the natural contraction of the blood vessel after the injury and changes in the plane of focus. Changes in the perfusion of physiological solutions, e.g., to remove extracellular Ca^2+^ or to add Ca^2+^ channel inhibitors/agonists, could introduce artifacts that affect the Ca^2+^ tracings (Bootman et al., 2013). Dye photobleaching during long-lasting fluorescence recordings and the inherent noise of optical and electronic instrumentation are also added to the Ca^2+^ signal and increase the difficulty of detecting true Ca^2+^ events.

This work focuses on discovering true Ca^2+^ events in *in situ* ECs by evaluating their ICPs during the Ca^2+^ response to mechanical injury. The ensuing increase in [Ca^2+^]_i_ at the wound edge is of paramount importance to promote wound repair (Jonkman et al., 2014; Moccia et al., 2014), and therefore, an automated software application to extract and measure the ICP from multiple cells would be helpful, e.g., to separate the endothelial Ca^2+^ responses that induce proliferation (i.e., oscillations) from those that stimulate migration (i.e., biphasic signals) (Noren et al., 2016).

## 2 Materials and methods

### 2.1 *In situ* endothelial cells

**FIGURE 1:**
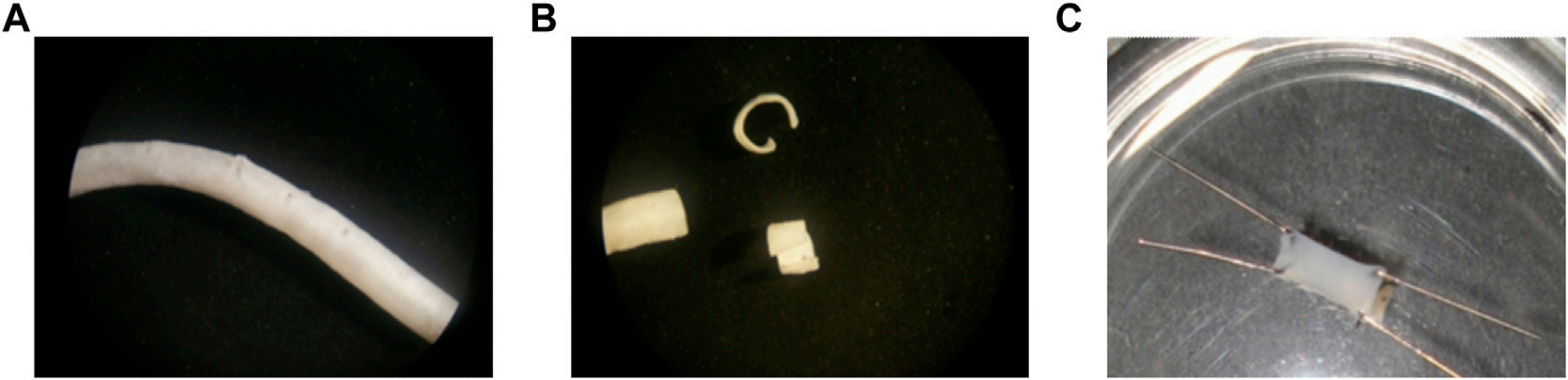
From left to right: **(A)** cleaned dissected aorta, **(B)** aorta cut in 5-mm-wide aortic rings (two of them carefully cut open), and **(C)** aortic strips with the intact endothelium fixed (with the luminal face up) to the bottom of a 35-mm diameter Petri dish covered by inert silicone by using four 0.4-mm diameter pins.

The intact endothelium was harvested from the thoracic and abdominal aorta of 1–3 month-old Wistar rats anesthetized with intraperitoneal ketamine–xylazine solution (0.2 mL per 100 g of weight). The aorta was first dissected and then perfused with physiological salt solution (PSS). Using a stereomicroscope (Nikon SMZ-2T, Tokyo, Japan), the connective and fatty tissues surrounding the aorta were removed. Subsequently, the aorta was cut into ∼5-mm-wide rings, stored in PSS at controlled room temperature (22°C–23 °C), and used within 5 h. Using a pair of microdissection scissors, the aortic rings were carefully cut open to obtain aortic strips with the intact endothelium. The aortic strips were loaded with 16 µmol Fura-2/AM for 60 min at room temperature, and then washed and fixed (with the luminal face up) to the bottom of a Petri dish covered by inert silicone (Sylgard ^®^ 184 Silicone Elastomer, Dow Corning, MI, United States) by using four 0.4-mm diameter pins. The procedure is shown in Figure 1. All the experimental procedures on the animals were performed according to protocols approved by the Animal Care and Use Committee of the Benemérita Universidad Autónoma de Puebla, identification code: BERRSAL71, 18-05-2017. Every effort was made to minimize the number of animals used and to ensure minimal pain and/or discomfort.

### 2.2 Solutions

The composition of the PSS expressed in mmol/L was NaCl (140), KCl (4.7), MgCl_2_ (1.2), glucose (6), CaCl_2_ (2.5), and HEPES (5). The solution was titrated to pH 7.4 with NaOH. All reagents were purchased from Sigma-Aldrich (Milan, Italy).

### 2.3 [Ca^2+^]_i_ measurements

As described previously (Berra-Romani et al., 2008; Berra-Romani et al., 2010; Berra-Romani et al., 2013), *in situ* ECs were loaded with 16-µmol Fura 2/AM (Molecular Probes Europe BV, Leiden, Netherlands) and visualized using an upright epifluorescence Axiolab microscope (Carl Zeiss, Oberkochen, Germany), equipped with a Zeiss ×40 Achroplan objective (water-immersion, 2.05-mm working distance, 1.0 numerical aperture). To monitor the changes in [Ca^2+^]_i_, ECs were excited alternately at 340 and 380 nm and the emitted light was detected at 510 nm. A neutral density filter (optical density = 1.0) was coupled to the 380-nm filter to approach the intensity of the 340-nm light. A round diaphragm was used to increase the contrast. A filter wheel (Lambda 10, Sutter Instrument, Novato, CA, United States) commanded by a computer was positioned alternately along the optical path of the two filters that allowed the passage of light at 340 and 380 nm, respectively. Custom software, working in the LINUX environment, was used to drive the camera (Extended-ISIS Camera, Photonic Science, Millham, United Kingdom) and the filter wheel. The acquisition frequency was 0.33 Hz, and the fluorescence images obtained at 340 nm and 380 nm were stored in the hard disk, and data analysis was conducted later using MATLAB^®^ R2020a.

ROIs were placed automatically as rectangular bounding boxes, each enclosing a single cell. Time-dependent signals, also known as ICPs, were acquired as the mean of the pixel-to-pixel ratio of the emissions at 340 and 380 nm (340/380) within each ROI, frame to frame throughout all the image sequences. An increase in [Ca^2+^]_i_ causes an increase in the 340/380 ratio.

For each experiment, ICPs were extracted for all the detected cells using the automated cell tracking system reported by Sanchez-Tecuatl et al. (2018; the reliability limit parameter was set to RL = 5. Only those cells with “ONLINE” status, i.e., reliably tracked, were included in the peak detection analysis. Table 1 shows the number of ICPs per experiment. ICPs were smoothed with a third-order polynomial Savitzky–Golay filter with a 11-sample window, as described by Lee et al. (2018), before the Ca^2+^ true event analysis.

**TABLE 1:** Experiment data, segmented cells, and online cells stand for those that were properly tracked across all frames.

Sample ID	#Segmented cells	#Online cells	#Frames	#GT true events
n1_s1	211	114	233	435
n1_s2	230	131	250	371
n1_s3	266	153	310	162
n1_s4	224	115	190	231
n1_s5	247	187	250	315
n2_s1	206	125	216	169
**Total**	**1,384**	**825**	**1,449**	**1,683**

Bold values summarize and highlight the total amount of items per column.

**FIGURE 2:**
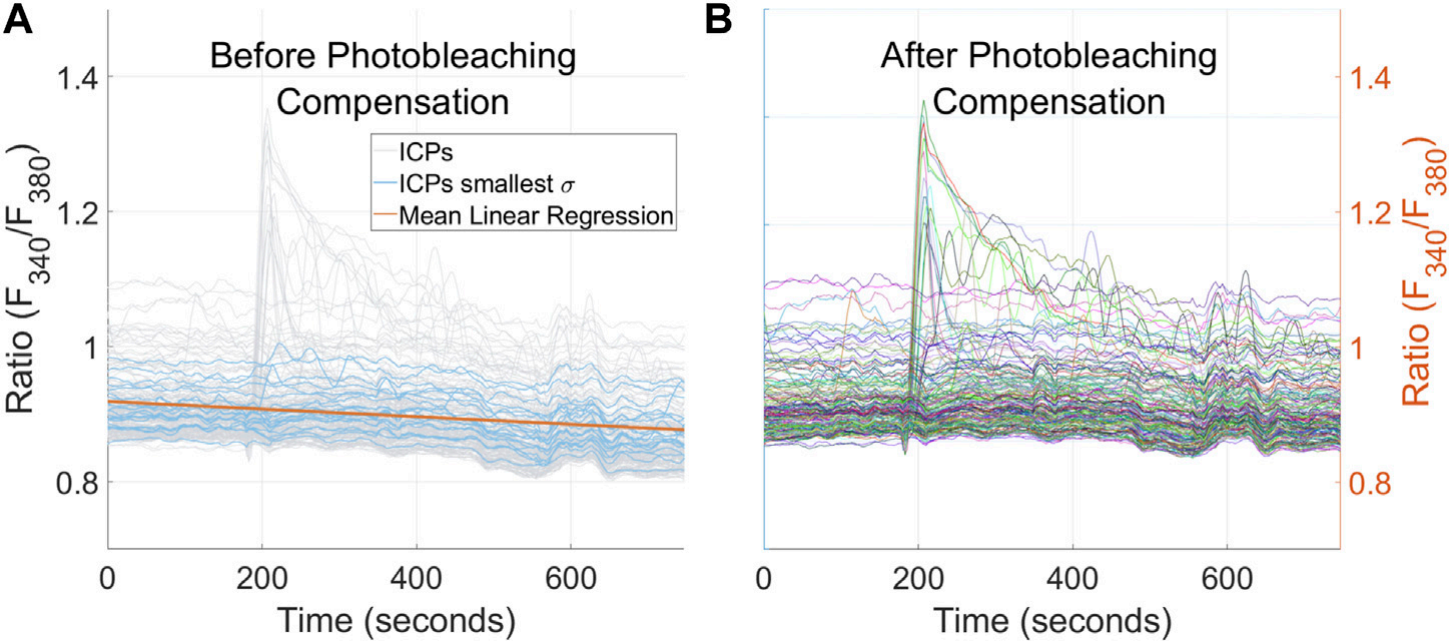
**(A)** ICPs before photobleaching compensation: all ICPs are plotted in gray, the 20 ICPs with the smallest standard deviation are in blue, and the mean of their linear regressions is in orange. **(B)** ICPs after slope compensation. The distance between the ICPs and background grid should be noted. Ratios are represented as arbitrary units (a.u.).

Fura-2-loaded cells exposed to excitation light for a long time may be affected by photobleaching, which is seen as a fluorescence decay that decreases the magnitude of intracellular Ca^2+^ profiles, i.e., a negative slope. This problem can affect further classification of ICPs. Thus, a compensation based on the linear regression slope was applied to the intracellular Ca^2+^ signals. For this reason, linear regression of the 20 ICPs with the smallest standard deviation was calculated and averaged, as shown in Figure 2.

### 2.4 Mechanical disruption of *in situ* endothelial cells

**FIGURE 3:**
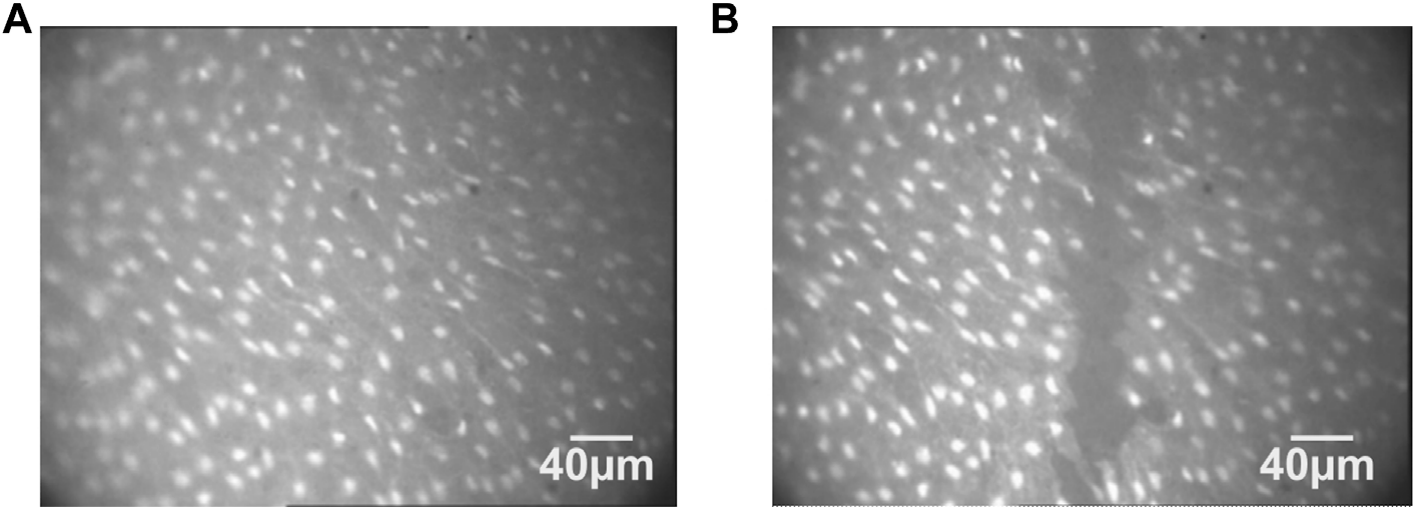
Fluorescence images of the endothelium cells before **(A)** and after **(B)** being injured with a central and vertical lesion.

As shown in several studies (Berra-Romani et al., 2008; Berra-Romani et al., 2012), the aortic endothelium was injured under microscopic control by means of a glass micro-pipette with a tip of about 30-µm diameter, driven by an XYZ hydraulic micromanipulator (Narishige Scientific Instrument Lab., Tokyo, Japan). The microelectrode was first positioned almost parallel and very near to the endothelium surface. Then, it was moved downward along the *z*-axis, as shown in Figure 3. Images of Fura-2-loaded ECs were taken before the lesion in order to identify the cells facing the injury site.

### 2.5 Ground truth segmentation

To assess the performance of the proposed approach for intracellular Ca^2+^ true event detection, a Ca^2+^ event ground truth (CE-GT) set of relevant [Ca^2+^]_i_ events in the CPs was manually generated by two health science experts for all ICPs for each endothelium sample. First, each expert independently generated the CE-GT events’ sets; then, a final CE-GT was determined by consensus of both experts. The total amount of true events pointed out for the six tissue samples was equal to 1,683. It is worth noting that not all the ICPs present true events (CE-GT marks) due to the heterogeneous pattern between individual cells in the injured *in situ* endothelium (Berra-Romani et al., 2008). In addition, CE-GT was generated by visual inspection and manual pointing through mouse positioning on continuous ICP plots of discrete data: therefore, some markers may be placed around the discrete data samples, which might lead to some inaccuracies.

**FIGURE 4:**
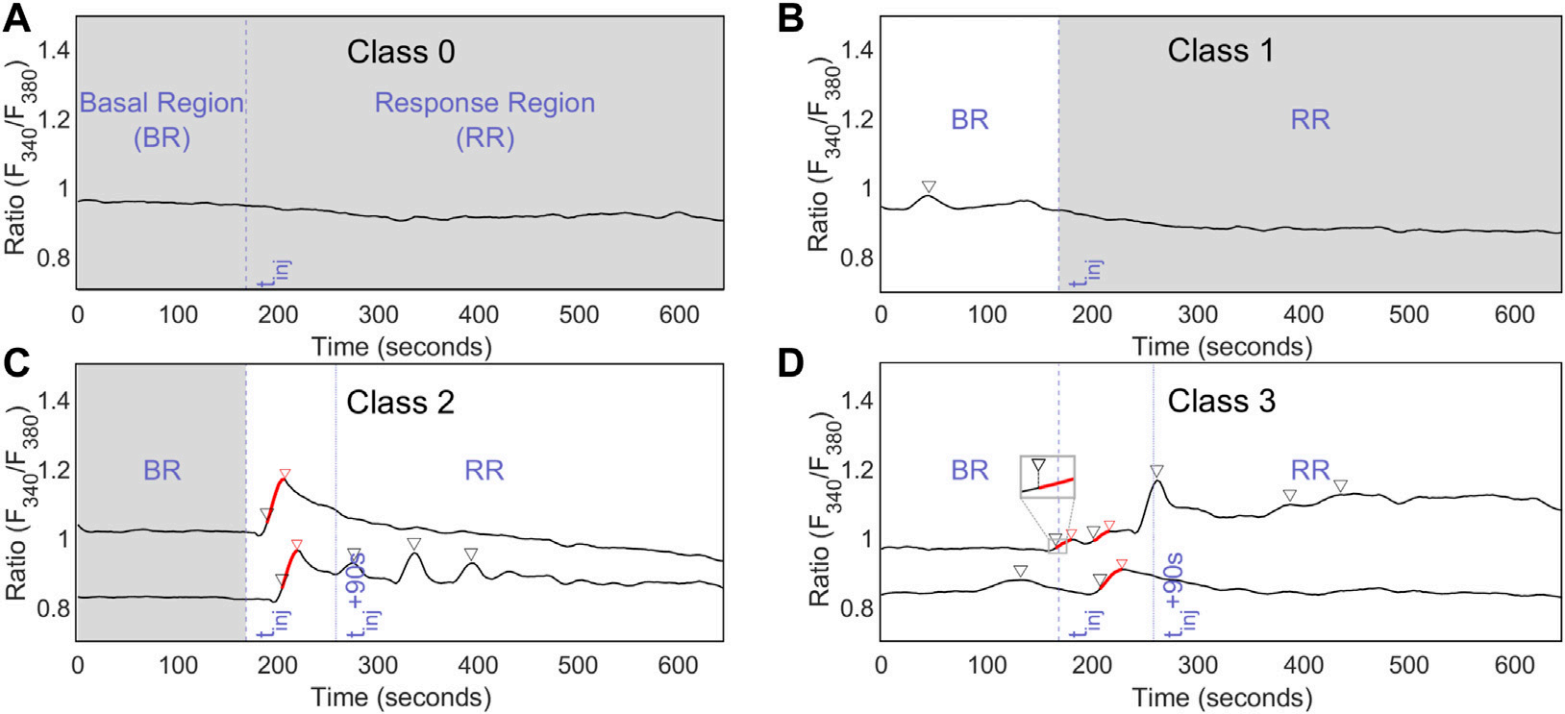
Classification ground truth (C-GT) criteria and class examples. The basal region (BR) and response region (RR) were defined by injury start time t_inj_. Regions with Ca^2+^ true events are highlighted with a white background, while regions without Ca^2+^ events are highlighted with a gray background, based on class definition. Classes 0, 1, 2, and 3 are shown in **(A–D),** respectively. CE-GT points are depicted with triangular markers; the red ones in **(D)** were left shifted to approximately 20% of their prominence value.

A second GT set (C-GT) was created to classify the ICPs according to the time of occurrence of the Ca^2+^ true events, i.e., whether Ca^2+^ activity could be recorded before and/or after the mechanical stimulus (see Table 2). Basal regions (BRs) and response regions (RRs) were defined as the portion of ICPs before and after the injury stimulus, respectively, as shown in Figure 4. Four classes arose from the binary detection of Ca^2+^ true events in each region; this C-GT included a total of 825 ICPs (six tissue samples).

**TABLE 2:** Binary-coded classes. ✓: true events present. ✗: no true events present.

Class	Response region (RR)	Basal region (BR)
0	✗	✗
1	✗	✓
2	✓	✗
3	✓	✓

Due to the presence of a fast increase in [Ca^2+^]_i_ produced by the depletion of the main intracellular Ca^2+^ reservoir, the endoplasmic reticulum (ER), a 90-s window just after the mechanical stimulus was considered for classes 2 and 3. The CE-GT points within [t_inj_, t_inj_+90s] were set at the beginning of the event rise (around 20% of its prominence) rather than at its peak (see details of these situations in the CE-GT markers connected with the red line in Figure 4). The time window was determined experimentally. Then, C-GT was based on CE-GT.

### 2.6 Prominence thresholding

To detect a true Ca^2+^ event from an ICP, we define a left-prominence (LP) value in Eq. 1, where ${lmax}_{k}$ and ${lmin}_{k}$ stand for the *k*th local maximum and minimum, respectively.

$${LP}_{k}=\left|{lmax}_{k}-{lmin}_{k}\right|.$$ (1)

**FIGURE 5:**
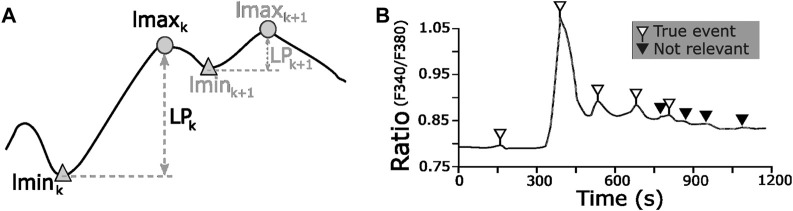
Left prominence value as a numerical tool to identify Ca^2+^ true events. **(A)** LP definition, index k is used to depict coupled local maximum and minimum; maxima without left minima at the boundaries are ignored. **(B)** Representative CP with blank and solid flags pointing out the true and non-relevant Ca^2+^ events, respectively.

As shown in Figure 5, a **local maximum** ( $\left.{lmax}_{k}\right)$ follows a **local minimum** ( $\left.{lmin}_{k}\right)$ , i.e., ${lmax}_{k}$ is preceded by ${lmin}_{k}$ , ${lmax}_{k+1}$ by ${lmin}_{k+1}$ , and so on.

**FIGURE 6:**
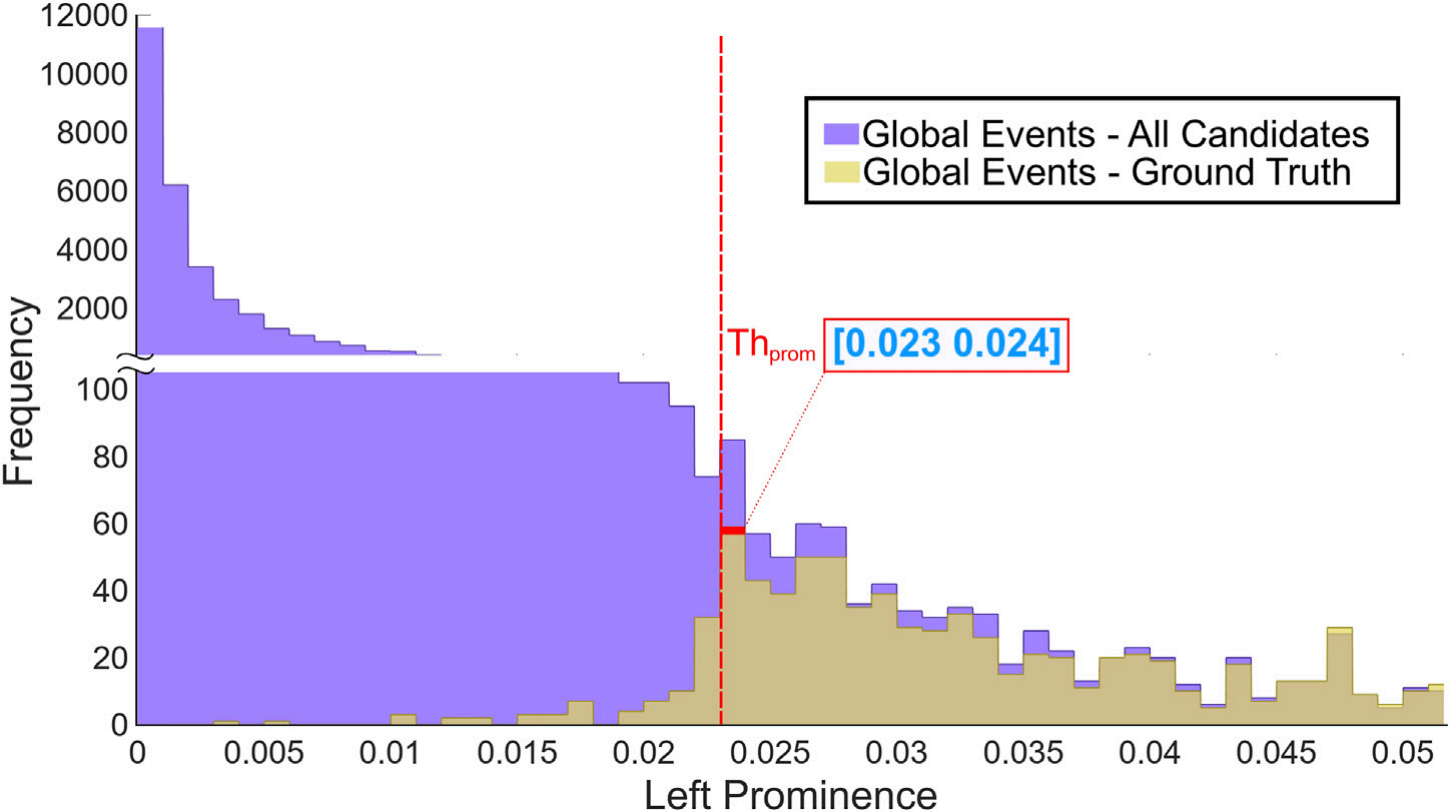
Overlapping histograms’ snippet of left prominence values. The LP overall event candidates are shown in purple, whereas ground truth Ca^2+^ true events are depicted in yellow, and the intersection between both histograms is shown in dark yellow. The red marker and data tip show the range (0.023,0.024) of the maximum frequency for ground truth LP. The frequency differences between event candidates and ground truth are markedly larger for LP < 0.02303.

The LP values of all the event candidates, i.e., the associated local maxima and minima for each ICP, were computed. Then, an LP threshold value was determined based on the most frequent small true events of CE-GT. Figure 6 shows a snippet of the overlapping histograms of LP values for CE-GT and event candidate sets, and the red dot data tip points out the LP range of maximum frequency for the CE-GT set, 0.023–0.024 a. u. The difference between histograms markedly increases for smaller LP values (<0.023), which means that LP values below the CE-GT set mode contains much more non-true events; this is the applicable criteria to select a good threshold candidate for new datasets without ground truth availability. Then, the selected range to assess our approach was 0.020–0.026, to include the CE-GT mode bar and its more relevant left and right portions.

### 2.7 True Ca^2+^ event detection

The detection of true Ca^2+^ events was implemented in MATLAB^®^ R2020a, and a general algorithm is shown in **Listing 1**. Functions with different possible implementations for the programmer were written in bold and pascalcase style^1^, arguments given in parenthesis. Snakecase style^2^ was assigned to variables; the element’s indices are given in parenthesis. Generic and self-explanatory names were assigned to both functions and variables. Notice that subindex i iterates across the ICPs, whereas the subindex *k* does across the couples of local maxima and minima, i.e., the candidate events.

Algorithm 1 Identification of true Ca^2+^ events (tce) in an intracellular Ca^2+^ profile (icp) using left prominence thresholding.

**DATA**:
icp: intracellular Ca^2+^ profiles
lp_threshold: user defined left prominence threshold

**RESULT**:
tce: true Ca^2+^ events

**MAIN PROGRAM**:
FOR EACH icp_i_

local_max_index = **FindLocalMaximumsLocation**(icp_i_)
local_min_index = **FindLocalMinimumsLocation**(icp_i_)
FOR EACH local_max_k_

local_min_index_k_=**FindClosestLeftMinIndex**(local_min_index,local_max_index_k_)
tce_left_prominence_i,k_ = icp(local_max_index_k_)- icp(local_min_index_k_)
END
tce_i_ = tce_left_prominence_i_ >= lp_threshold
END

Listing 1. Pseudocode to detect true Ca^2+^ events based on left prominence thresholding

As mentioned previously, ICPs are prone to present complex structures and undesirable artifacts due to mechanical stimuli, extracellular perfusion solution change, or natural tissue contraction that may move fluorescence images out of focus. Therefore, manual focus adjustments are required along the experiment, thereby resulting in additional peaks in the ICPs that hinder the detection of relevant Ca^2+^ true events. The effects of the mechanical injury also impact the procedure of cell tracking that produces the difference between segmented cells and online cells shown in Table 1. More details are reported in previous studies (Jonkman et al., 2014; Sanchez-Tecuatl et al., 2018).

**FIGURE 7:**
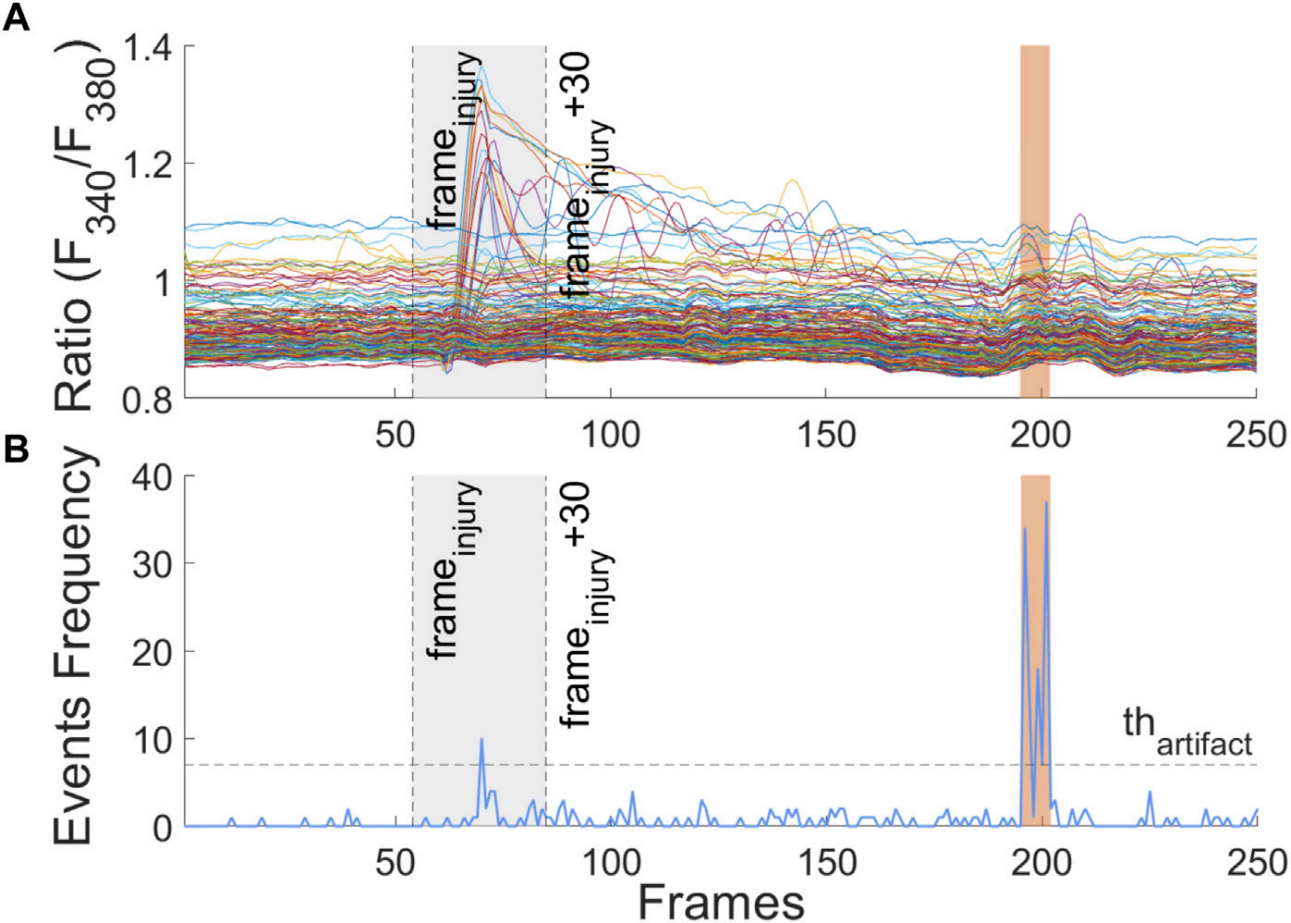
**(A)** Multiple ICPs are shown, the artifact region that matches with the sudden increase in [Ca^2+^]_i_ is orange-shaded. (B) Events' frequency used to detect and discard artifacts through thresholding. The gray shaded region corresponds to the artifact detection disabled time window, just after mechanical stimulus.

When an artifact appears, it impacts many cells in the visual field. Mechanical artifacts can, therefore, be added to Ca^2+^ tracing, but they do not have to be classified as Ca^2+^ true events. An approach was designed to detect and remove these artifacts based on the frequency of Ca^2+^ events above the LP threshold at a given time point. An event frequency threshold ${th}_{art}$ was defined and used to locate the artifact regions across ICPs, as shown in Figure 7. It should be noted that just after the mechanical stimulation, the number of cells that respond is comparable to the number of cells impacted by the artifact and a genuine Ca^2+^ event may be erroneously discarded, while false negatives may appear. For this reason, the artifact detection was disabled during a 30-frame window (90 s) starting by the injury time that was calculated, shown in Eq. 2, as the shifted maximum of the frame-to-frame sum of thresholded first-order difference of each ICP, as shown in Eq. 2.

$${frame}_{injury}=\max\left(\sum_{j=1}^{n_{profiles}}\left[{ICP}_{j}^{\prime }\left(i\right)>{th}_{diff}\right]\right)-kT_{s},$$ (2)

where ${th}_{diff}=0.040$ and k = 10 were determined experimentally, $T_{s}=3s$ is the temporal image sample period, and *i* and *j* operate across ICPs and frames, respectively. All Ca^2+^ events inside the detected artifact regions were ignored for further analysis or classification processes.

### 2.8 ICP classification

Once the event candidates were thresholded with the left prominence (LPT) criterion and artifacts were discarded (AD) from the event candidates, the remaining event candidates were considered Ca^2+^ true events. As per C-GT, the Ca^2+^ true events within the interval [t_inj_, t_inj_+90s] were left shifted by our classifier as well to the 0.2*lp value of the respective event. Finally, ICPs were classified (ICP_C) based on their time location, as shown in Table 2 and Figure 4.

## 3 Results

### 3.1 True Ca^2+^ event detection

**FIGURE 8:**
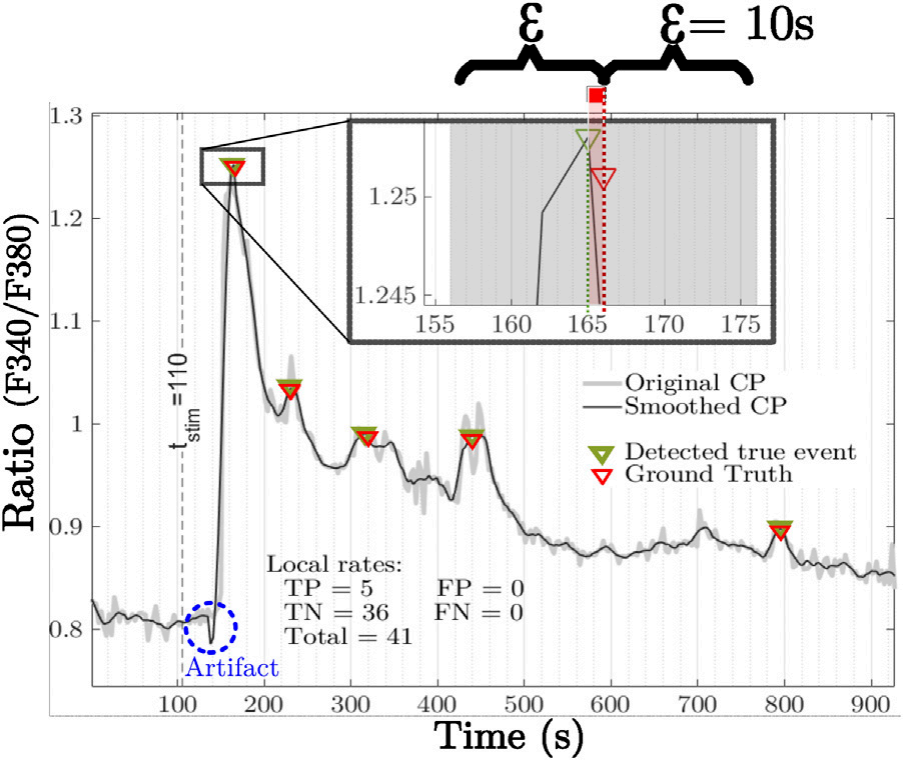
An actual ICP is shown with original and smoothed signals overlapped. Red and green marks represent the ground truth and detected true events (by our approach), respectively. The inset shows the neighborhood, gray area, of 2σ introduced to quantify coincidences of the true events detected with CE-GT marks. It should be noted that inaccuracy is mitigated as five coincidences are reported as true positives (TP), and 36 more candidates were discarded by the LP threshold of 0.024 and quantified as true negatives (TN). In this case, there are neither false-positives nor false-negatives (FP and FN, respectively). The total sum of all local rates (FP and TN) must, therefore, match with the total event value provided by the analysis. Mechanical stimulus was applied at $t_{stim}$ = 110 s. The depicted signal response of the Ca^2+^ profile is delayed due to the natural propagation time, depending on the cell position referenced to the injury site. The fluorescence artifact, due to the mechanical injury of the intact endothelium, is marked by the blue dashed circle.

Since the CE-GT was obtained manually, using a pc-mouse as the pointer, there is an inherent inaccuracy between the selected CE-GT points and the maxima of the actual true events. Therefore, a symmetric neighborhood of size 2 epsilon (units in seconds) was used as a tolerance to quantify the coincidences between the CE-GT and the true Ca^2+^ events resulting from our approach, and thus, evaluate its performance (Figure 8 **)**.

Performance measurements of Ca^2+^ true events’ identification are shown in Table 3 with *ε* = 10 (approximately three samples’ length) for our approach in the left prominence section. Results with ε = 0 for each ICP baseline noise standard deviation (σ_bn_) thresholding are reported in the second section, i.e., each ICP determined its own threshold. Meanwhile, an approach based on the minimum baseline noise standard deviation of the corresponding dataset in the last section (σ_bn_ lowest noise), i.e., the ICP with the lowest baseline noise, determined the threshold for the other ICPs. Comparison between approaches is addressed in the Discussion section. The maximum, minimum, and mean values obtained for the six datasets are reported for different threshold values.

**TABLE 3:** Summary of the performance results of Ca^2+^ events thresholding for two different approaches: the first one was based on the left prominence value, our approach, and the second and third approaches were based on n-times the standard deviation of baseline noise ( $\sigma_{bn}$ ) of each profile and of the profile with the least noise, respectively. Highest maxima and lowest minima are gray-shaded.

	Threshold	Sensitivity			Specificity			Accuracy		
		Max	Min	Mean	Max	Min	Mean	Max	Min	Mean
Left prominence	0.020	0.9993	0.9962	0.9981	0.9013	0.7818	0.8486	0.9951	0.9579	0.9863
	0.021	0.9995	0.9949	0.9979	0.9434	0.8034	0.8830	0.9968	0.9645	0.9893
	0.022	0.9995	0.9942	0.9978	0.9856	0.8275	0.9139	0.9977	0.9708	0.9917
	0.023	0.9990	0.9932	0.9964	0.9849	0.8302	0.9182	0.9978	0.9697	0.9909
	0.024	0.9986	0.9870	0.9946	0.9946	0.8337	0.9252	0.9977	0.9669	0.9903
	0.025	0.9983	0.9831	0.9937	0.9945	0.8373	0.9314	0.9974	0.9656	0.9899
	0.026	0.9981	0.9807	0.9924	0.9944	0.8403	0.9355	0.9973	0.9648	0.9892
σ_bn_	1	1.0000	0.9957	0.9982	0.5682	0.5165	0.5378	0.7904	0.6628	0.7106
	3	0.9978	0.9813	0.9932	0.6820	0.5543	0.6130	0.9740	0.8533	0.9174
	4	0.9979	0.9778	0.9924	0.7511	0.5761	0.6717	0.9898	0.8916	0.9485
	7	0.9960	0.8904	0.9736	0.9613	0.6983	0.8411	0.9950	0.8888	0.9696
	9	0.9945	0.8911	0.9698	1.0000	0.7446	0.8998	0.9944	0.8954	0.9695
	10	0.9944	0.8872	0.9673	1.0000	0.7509	0.9218	0.9943	0.8937	0.9680
	13	0.9908	0.8445	0.9549	1.0000	0.7976	0.9478	0.9908	0.8514	0.9556
σ_bn lowest noise_	1	1.0000	0.9942	0.9990	0.5412	0.5079	0.5238	0.5652	0.4130	0.5210
	3	1.0000	0.9974	0.9987	0.5758	0.5215	0.5483	0.8392	0.6343	0.7820
	4	1.0000	0.9974	0.9984	0.5994	0.5333	0.5637	0.8986	0.6894	0.8392
	7	0.9988	0.9971	0.9982	0.7109	0.5723	0.6347	0.9717	0.7913	0.9254
	9	0.9992	0.9971	0.9984	0.8238	0.6136	0.7110	0.9873	0.8334	0.9533
	10	0.9991	0.9973	0.9982	0.8971	0.6300	0.7558	0.9912	0.8495	0.9622
	13	0.9990	0.9907	0.9966	0.9945	0.6664	0.8738	0.9978	0.8937	0.9769

The aforementioned results show that our approach properly identifies the Ca^2+^ true events with a sensitivity range of [0.99, 0.98], whereas its specificity range [0.99, 0.78] shows good performance on discarding the non-relevant activity. It should be noted that accuracy is in the range [0.99, 0.95] across the six datasets. All the values were rounded to two decimals.

### 3.2 ICP classification

**FIGURE 9:**
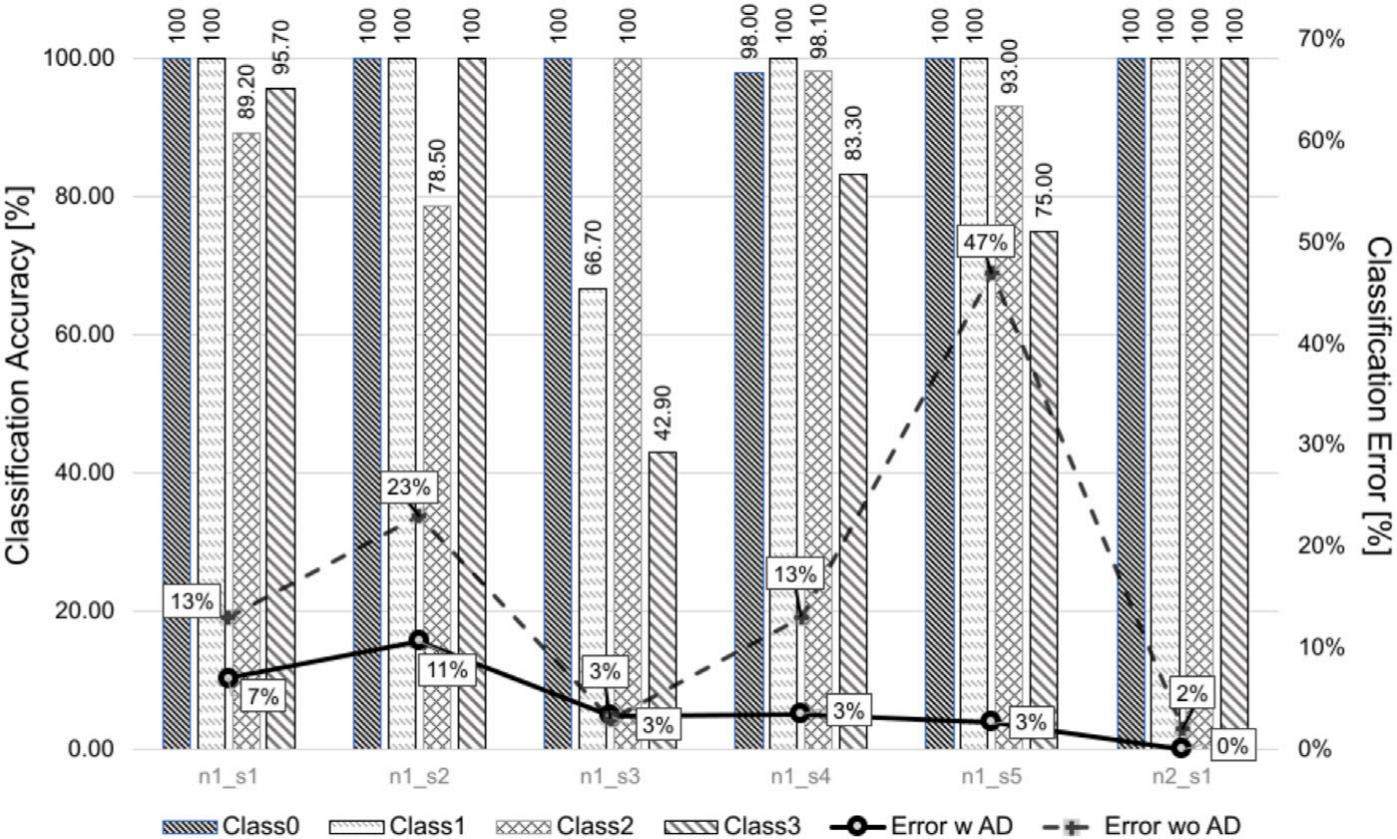
Accuracy of ICPs’ classification grouped per tissue sample. Bars represent accuracy classification values, and dashed and solid lines represent the error percentage without and with artifact detection, respectively. Accuracy values relative to the number of elements of each class are shown inside bars.

Performance assessment of the scheme described in **subsection 2.8** (LPT + AD + ICP_C) was carried out based on the C-GT (Figure 9). Accuracy results average 92.52%, including all classes for all tissue samples. Error percentage values are shown in solid purple, whereas the dashed line shows the error percentage for a scheme without artifact detection, i.e., LPT + ICP_C only. It should be noted that errors are markedly reduced with AD stage, reducing errors up to 44%.

Class 0 was the best identified, ${\bar{acc}}_{C0}=99.67\%$ , which sets a good basis for responsive and non-responsive cells’ classification. The remaining classes showed an accuracy reduction ${\bar{acc}}_{C1}=94.45\%$ , ${\bar{acc}}_{C2}=93.13\%$ , and ${\bar{acc}}_{C3}=82.82\%$ ; it should be noted that the worst accuracy cases are those with a few elements in class; thus, a single ICP wrongly sorted reduced accuracy values.

## 4 Discussion

Several approaches have been developed to understand how distinct intracellular Ca^2+^ signatures regulate different physiological processes. On one hand, studying the intracellular patterns of Ca^2+^ pulses or wave propagation only provides a single-cell perspective (Greensmith, 2014; Lee et al., 2018; Leigh et al., 2020). On the other hand, high-throughput tools are required to reliably automate the processing of a huge amount of Ca^2+^ data generated by the analysis of hundreds of cells at the tissue level (Francis et al., 2012; Sanchez-Tecuatl et al., 2018; Shah et al., 2020). In this context, this study illustrated and validated a novel approach to detect the Ca^2+^ true events, which effectively identifies meaningful structures and discards the non-relevant structures in the ICPs.

Many analysis tools use *n*-times the standard deviation of the baseline fluorescence noise for individual cells as the threshold value to enhance true event detection and identify the relevant Ca^2+^ activity in the ICPs. Some of these approaches calculate the baseline noise of ICP regions prior to an expected and clearly marked Ca^2+^ event (Wilson et al., 2016; McCarron et al., 2017; Lee et al., 2018; McCarron et al., 2019; Buckley et al., 2021) and some others determine the baseline by averaging ICP portions with the lowest noise (Wilson et al., 2020). Usually, the Ca^2+^ response is elicited by chemical stimulation, which is less prone to introduce fluorescence artifacts than the mechanical injury maneuver described in the present study. Whole ICP baselines of each dataset were considered for the performance assessment of thresholding methods. This comparison showed that undesired fluorescence artifacts due to the experimental procedures negatively impact the performance of methods based on the standard deviation of baseline noise since outlier values may increase the standard deviation and the number of false positives.

The assessment shown in Table 3 indicates that our approach to identify Ca^2+^ true events provides better performance ranges than baseline noise standard deviation thresholding methods. Sensitivity showed a competitive range, 0.01, compared with σ_bn_ and σ_bn lowest noise_. In terms of specificity, our method showed a less spanned range, 0.21, than the others, 0.48, which means that our approach identifies true negatives better than 2.0x of σ_bn_ and σ_bn lowest noise_. Although maxima values of accuracy are very close between three thresholding methods, our proposal shows significant smaller dispersion, 0.0399, than 0.3322 and 0.5848 for σ_bn_ and σ_bn lowest noise_, respectively. This corresponds to an improvement above 14x. The three main highlights of our proposal are 1) sturdiness, since it can work regardless of the length of the ICP basal stage as left prominence is defined based on couples of local maxima and minima: this approach is applicable even in datasets where fluorescence artifacts can appear due to mechanical maneuvers generating tissue displacement, cell movement, and blurred optical field (see the blue dashed circle in Figure 8); 2) simplicity, since it is not required to select low-noise regions in ICPs across datasets, which may be a tedious supervised task; and 3) stability, since in view of the variability of the datasets, it provided less dispersion in its performance ranges than other approaches.

On top of the LP thresholding approach, a method was proposed and implemented to detect and discard undesirable artifacts of ICPs. This method relies on the frequency per frame of event candidates resulted from the LP thresholding process. Alongside, a 4-class classifier was implemented based on true events’ identification and their time location. Performance assessment showed 92.52% of average accuracy (4 classes) with outstanding capability to split datasets into responsive and non-responsive cells, while classes with Ca^2+^ true events showed a competitive average accuracy of 90.1% (classes 1, 2, and 3). Our proposal correctly classified 789/825 ICPs and showed better performance than the 4-class scheme reported by Payne et al. (2005), which scored 580/823. Classes may be redefined according to the analysis purpose in turn.

Of note, our method would also work with healthy (i.e., control) and pathological (e.g., diabetes, hypertension, atherosclerosis, obesity, and aging) ECs, thereby presenting the potential to be extremely helpful to analyze whether and how endothelial dysfunction is associated with deregulated intracellular Ca^2+^ activity. Furthermore, this approach would also be effective in analyzing the ICPs generated by other cell types, other dyes, or even signals recorded by chemical stimulation. It can handle any single-defined acquisition frequency signals for a given experiment, which may allow microdomain events’ detection on given proper experimental data; the system requires specifying the defined acquisition period to display signals in a proper time scale. Different experimental protocols may require custom pre- or post-processing algorithms to remove artifacts.

## 5 Conclusion

In conclusion, we have presented a proposal to assist with the bulk analysis of ICPs through the detection of Ca^2+^ true events, removal of undesired artifacts, and event time location-based classification. This approach proved to be more precise, accurate, and reliable than previous proposals in performing automatic ICP detection, is suitable for cultured cells, and fully addressed all the challenges imposed by mechanical stimulation of the naïve endothelium. In addition to the Ca^2+^ signals evoked by mechanical stimulation, this approach would be suitable to detect ICPs induced by other modes of mechanical stimulation, such as changes in pulsatile pressure of shear stress. In addition, it could be efficiently employed for the automatic detection of ICPs evoked by neurohumoral stimulation. Finally, by enabling a more accurate evaluation of the ICPs occurring at the injury site in *in situ* endothelial cells, this approach could be instrumental in evaluating the role of intracellular Ca^2+^ signaling in endothelium regeneration processes and vasomotion.

## Data Availability

The raw data supporting the conclusion of this article will be made available by the authors, without undue reservation.

## Footnotes

1 Pascalcase is a naming convention where the first letter of each word in a compound word is capitalized.

2 Snakecase is a naming convention where each space is replaced by an underscore (_) character, and the first letter of each word is written in lowercase.

## Supplementary material

SUPPLEMENTARY VIDEO S1 Increase in Fura-2 fluorescence intensity in response to mechanical stimulation.
